# Cardiac structural and functional abnormalities in epilepsy: A systematic review and meta‐analysis

**DOI:** 10.1002/epi4.12692

**Published:** 2023-01-31

**Authors:** Zining Liu, Peravina Thergarajan, Ana Antonic‐Baker, Zhibin Chen, Paul B. Sparks, Natasha A. Lannin, Patrick Kwan, Nigel C. Jones, Pablo M. Casillas‐Espinosa, Piero Perucca, Terence J. O'Brien, Shobi Sivathamboo

**Affiliations:** ^1^ Department of Neuroscience, Central Clinical School Monash University Melbourne Victoria Australia; ^2^ Department of Medicine, The Royal Melbourne Hospital The University of Melbourne Melbourne Victoria Australia; ^3^ Department of Cardiology The Royal Melbourne Hospital Parkville Victoria Australia; ^4^ Alfred Health Melbourne Victoria Australia; ^5^ Department of Neurology The Royal Melbourne Hospital Parkville Victoria Australia; ^6^ Department of Neurology Alfred Health Melbourne Victoria Australia; ^7^ Bladin‐Berkovic Comprehensive Epilepsy Program, Department of Neurology Austin Hospital Heidelberg Victoria Australia; ^8^ Department of Medicine (Austin Health), Epilepsy Research Centre The University of Melbourne Heidelberg Victoria Australia

**Keywords:** cardiac, epilepsy, seizures, SUDEP

## Abstract

**Objective:**

Epilepsy is associated with an increased risk of cardiovascular disease and mortality. Whether cardiac structure and function are altered in epilepsy remains unclear. To address this, we conducted a systematic review and meta‐analysis of studies evaluating cardiac structure and function in patients with epilepsy.

**Methods:**

We searched the electronic databases MEDLINE, PubMed, COCHRANE, and Web of Science from inception to 31 December 2021. Primary outcomes of interest included left ventricular ejection fraction (LVEF) for studies reporting echocardiogram findings and cardiac weight and fibrosis for postmortem investigations. Study quality was assessed using the National Heart, Lung, and Blood Institute (NHLBI) assessment tools.

**Results:**

Among the 10 case‐control studies with epilepsy patients (n = 515) and healthy controls (n = 445), LVEF was significantly decreased in epilepsy group compared with controls (MD: −1.80; 95% confidence interval [CI]: −3.56 to −0.04; *P* = 0.045), whereas A‐wave velocity (MD: 4.73; 95% CI: 1.87‐7.60; *P* = 0.001), E/e' ratio (MD: 0.39; 95% CI: 0.06‐0.71; *P* = 0.019), and isovolumic relaxation time (MD: 10.18; 95% CI: 2.05‐18.32; *P* = 0.014) were increased in epilepsy, compared with controls. A pooled analysis was performed in sudden unexpected death in epilepsy (SUDEP) cases with autopsy data (n = 714). Among SUDEP cases, the prevalence of cardiac hypertrophy was 16% (95% CI: 9%–23%); cardiac fibrosis was 20% (95% CI: 15%–26%). We found no marked differences in cardiac hypertrophy, heart weight, or cardiac fibrosis between SUDEP cases and epilepsy controls.

**Significance:**

Our findings suggest that epilepsy is associated with altered diastolic and systolic echocardiogram parameters compared with healthy controls. Notably, SUDEP does not appear to be associated with a higher incidence of structural cardiac abnormalities, compared with non‐SUDEP epilepsy controls. Longitudinal studies are needed to understand the prognostic significance of such changes. Echocardiography may be a useful noninvasive diagnostic test in epilepsy population.

**Key Points**

- This systematic review and meta‐analysis reported that multiple echocardiogram parameters were altered in people with epilepsy compared healthy controls
- SUDEP was not found to be associated with a higher incidence of structural cardiac abnormalities, compared with non‐SUDEP epilepsy controls
- Echocardiography may potentially be an important cardiac diagnostic tool in epilepsy to optimize clinical management of these patients

## 1 INTRODUCTION

Epilepsy is one of the most common neurological diseases, affecting approximately 70 million people worldwide.^1^ Epilepsy is associated with an increased risk of cardiovascular disorders including coronary heart disease, hypertension, and myocardial infarction.^2^ Multiple studies have demonstrated changes in cardiac structure and function in patients with epilepsy, which may form the substrate for the arrhythmogenesis of lethal tachyarrhythmias.^2^ Underlying cardiovascular disease is often predictive of ventricular tachyarrhythmias and cardiac arrest in people with epilepsy,^3^ and it is a major cause of premature mortality in epilepsy.^4^

Recurrent epileptic seizures, particularly tonic‐clonic seizures, commonly result in cardiac stress and ischemia, which may result in acute or chronic myocardial injury and autonomic dysfunction.^5^, ^6^, ^7^, ^8^ This has led to the emerging concept of the “epileptic heart,” which is hypothesized that damage to the heart is a result of catecholamines toxicity and hypoxemia in recurrent seizures, leading to electrical and mechanical dysfunction.^2^ Altered serological and electrocardiogram markers of myocardial injury and stress cardiomyopathy have also been observed following generalized convulsive seizures^9^ and status epilepticus (SE).^10^, ^11^ Epilepsy treatments such as enzyme‐inducing antiseizure medications (ASMs) or sodium channel modulators may also contribute toward this risk.^2^

Sudden unexpected death in epilepsy (SUDEP) is the leading cause of death directly related to epilepsy^12^ and ranks second only to stroke in terms of potential years of life lost.^13^ Proposed mechanisms include seizure‐induced respiratory and cardiac dysfunction, immediately following a tonic‐clonic seizure.^14^ However, the underlying mechanisms that lead to SUDEP are likely heterogeneous, including impaired cellular mechanisms that regulate serotonin and adenosine pathways and possibly genetic factors.^14^, ^15^ Altered cardiac function may be a biomarker for risk prediction in SUDEP.^16^ It has been suggested that people who die from SUDEP have increased prevalence of cardiac structural abnormalities,^17^ though these data are conflicting.^18^ Further complicating this is the overlap between sudden cardiac death (SCD) in epilepsy and SUDEP, the definitions of which are fraught with limitations and overlap.^2^

To address these uncertainties, we conducted a systematic review and meta‐analysis of all studies examining structural and/or functional cardiac changes in epilepsy. Furthermore, we examined whether epilepsy variables or major cardiovascular risk factors increase the risk of cardiac structural abnormalities in this population.

## 2 METHODS

The protocol for this review was developed and prospectively registered on the PROSPERO International Prospective Register of Systematic Reviews [CRD42020212849, 12 November, 2020] in accordance with PRISMA guidelines.^19^

### 2.1 Identification of studies

The electronic databases MEDLINE, PubMed, COCHRANE, and Web of Science were searched from inception to December 31, 2021. Searches were performed with the English‐language restriction using terms relating to “epilepsy,” “seizure,” “SUDEP,” “cardiac structure,” “cardiac fibrosis,” “echocardiography,” “postmortem,” and multiple variants of the terms (Appendix S1). Titles and abstracts (where available) were displayed and screened to identify relevant studies. Full paper copies of relevant studies were obtained, and their reference lists were screened.

### 2.2 Selection of studies

The studies were reviewed independently by two authors (ZL and PT) against a predetermined criteria, and conflicts were resolved by a third author (SS). Studies had to meet inclusion criteria, which consisted of a minimum of five participants with epilepsy reporting echocardiography or cardiac autopsy findings with numerical data. For studies measuring echocardiography, patients with known cardiovascular disease were excluded. We considered publications utilizing data from the same cohort as one study.

### 2.3 Study quality and risk of bias assessment

Risk of bias was assessed independently by two authors (ZL or PT, and SS) using the Quality Assessment Tools developed by the National Heart, Lung and Blood Institute (NHLBI).^20^ Disagreements were resolved by discussion with the full screening group. The NHLBI tools consist of 9‐14 items for different study types (observational cohort and cross‐sectional studies, case series studies, and case‐control studies); an overall quality score was calculated based on the sum of the dichotomized response (yes/no) to each item. Overall quality scores of <40% were considered low quality (rated “poor”).

### 2.4 Data extraction

Data extraction was conducted independently by two authors (ZL and PT) using a standardized electronic template and cross‐checked by another author (SS). Extracted information included patient demographics, epilepsy or seizure characteristics, cardiac and cardiovascular disease characteristics, echocardiography outcomes, and autopsy findings were provided (a full list of extracted information appear in Table S1). Where data were missing or incomplete, we contacted the respective corresponding study author by email to obtain the additional information.

### 2.5 Statistical analysis

We excluded studies that were rated “poor” based on the quality review in statistical analysis. Echocardiography measurements were only included in the meta‐analysis if a minimum of three studies reported the same measurement. Due to the high incidence of cardiovascular comorbidities, we excluded patients with SE from the meta‐analysis, but discussed the findings of these studies separately.^21^ Due to the small number of studies included in the meta‐analysis, we performed a random effect meta‐analysis with Hartung‐Knapp‐Sidik‐Jonkman approach to compare the mean difference (MD) of echocardiography measurements between people with epilepsy and healthy controls. Pooled estimates of the effects of cardiac hypertrophy, heart weight, cardiac interstitial or perivascular fibrosis were obtained, along with 95% confidence intervals.

The *I* ^2^ statistic was used to quantify the magnitude of between‐study heterogeneity. When there were at least five studies available, random effects meta‐regression was used to explore the source of heterogeneity and assess the moderating effect of factors. Variables include age, sex, BMI, and epilepsy characteristics (percentage of patients diagnosed with generalized epilepsy, year since epilepsy diagnosed, and percentage of patients taking monotherapy). The heterogeneity that can be explained by the moderator was quantified using *R* ^2^ statistic. Statistical significance level was set at *P* < 0.05. All statistical tests were conducted using *Stata* version 16 (StataCorp).

## 3 RESULTS

### 3.1 Study selection

**FIGURE 1:**
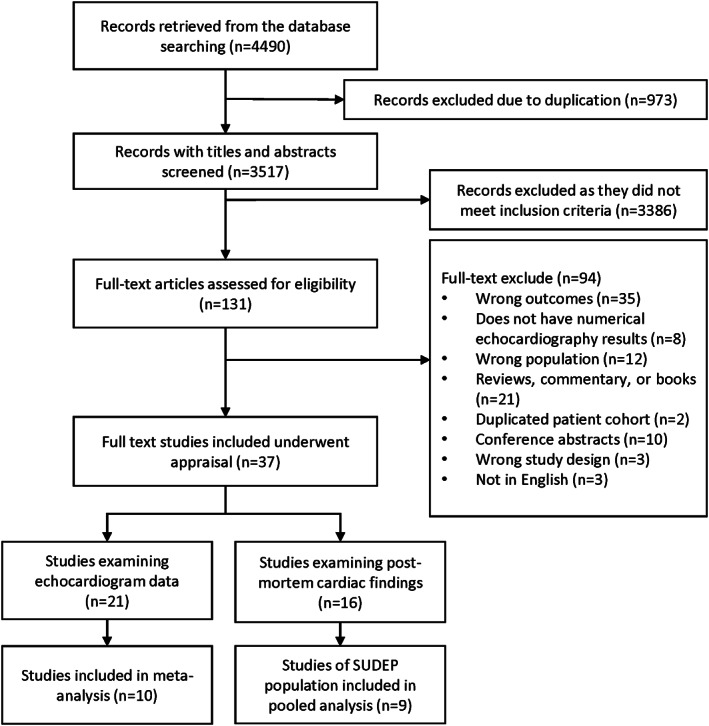
Selection of studies. Flowchart of the selection of studies examining cardiac structure and function in epilepsy from inception to 31 December, 2021.

Of the 4490 studies that were initially identified, 131 full‐text articles were independently reviewed for eligibility. Of these, 37 studies met our inclusion and exclusion criteria (Table S2). These comprised 21 studies reporting echocardiogram findings and 16 examining postmortem findings. The process of screening and reasons for exclusion are summarized in Figure 1.

### 3.2 Study quality assessment

Thirty‐seven studies underwent study quality assessment. The majority of included studies used a case‐control (n = 19) or cohort (n = 12) study design; four studies were cross‐sectional and two were case series (Table S2). Most studies had moderate risk of bias and were rated as “fair” quality. The average quality rating score was 55.7%, 53.5%, and 64.3% for case‐control studies, cohort and cross‐sectional studies, and case series studies, respectively. The results of quality appraisals for observational cohort and cross‐sectional studies, case‐control studies, and case‐series are summarized in Tables S3–S5. Six studies were excluded from meta‐analysis and pooled analysis as they did not meet criteria following quality appraisal (scoring < 40%).^22^, ^23^, ^24^, ^25^, ^26^, ^27^

### 3.3 Echocardiogram findings in epilepsy

Twenty‐one studies evaluated cardiac structural and/or functional in living patients with epilepsy using an echocardiogram (56.8%). Of these, eight studies examined adult populations, 11 studies examined pediatric populations, and two study examined both adult and pediatric populations. Criteria for exclusion of cardiovascular comorbidities were specified in 17 studies, but were not reported in the remaining four studies. The studies are summarized in Table S2.

### 3.4 Quantitative synthesis

#### 3.4.1 Meta‐analysis

The meta‐analysis included 10 prospective case‐control studies that performed standard transthoracic echocardiography measurements (Table S6) in people with epilepsy (n = 515) and healthy controls (n = 445); the characteristics of these studies are summarized in Table 1. Of 515 patients with epilepsy, 138 (26.8%) had generalized epilepsy, 140 (27.2%) focal epilepsy, and 10 (1.9%) epilepsy of unknown type; epilepsy classification was not reported in 227 (44.1%) cases. The mean epilepsy duration of these patients ranged from 2 to 22.5 years. Almost half of the patients (n = 245, 47.6%) reported a history of tonic‐clonic seizures. ASM use was reported in 289 (56.1%) patients, of whom 210 were on monotherapy and 79 were on polytherapy. Body mass index (BMI) was reported in seven studies, for which there were no significant differences between patients with epilepsy and controls.

**TABLE 1:** Characteristics of studies included in the meta‐analysis of echocardiography findings

Study	Population size (n)	Population	Study design	Age (y), mean ± SD	Female sex, n (%)	BMI (kg/m^2^), mean ± SD	Epilepsy classification, n (%)	Epilepsy duration (y), mean ± SD	History of tonic–clonic seizures, n (%)	ASM therapy, n (%)
e‐Ref #1, 2020	82 (41 epilepsy, 41 healthy controls)	Pediatric	Case‐control study, prospective	Epilepsy (10.1 ± 4.6), controls (10.1 ± 5.6)	Epilepsy 17 (41.5%), control 17 (41.5%)	NR	NR	7.8 (4.6–11.5)^a^	31 (75.6%)	Monotherapy 4 (9.8%), Polytherapy 37 (90.2%)
e‐Ref #4, 2021	60 (30 epilepsy, 30 healthy controls)	Adult	Case‐control study, prospective	Epilepsy (37.4 ± 11.2), controls (35.3 ± 9.3)	Epilepsy 18 (60%), healthy controls 18 (60%)	Epilepsy (37.4 ± 11.2), controls (25.5 ± 4.1)	Focal 30 (100%)	22.5 ± 10.7	5 (16.7%)	Polytherapy 24 (80%)
e‐Ref #9, 2013	180 (120 epilepsy, 60 healthy controls)	Pediatric and adult	Case‐control study, prospective	Epilepsy (25.2 ± 9.3), controls (27.3 ± 7.5)	Epilepsy 44 (36.7%), healthy controls 21 (35.0%)	Epilepsy (26.9 ± 6.3), controls (25.1 ± 9.2)	NR	Newly diagnosed	120 (100%)^b^	NR
e‐Ref #10, 2014	88 (52 epilepsy, 36 healthy controls)	Pediatric	Case‐control study, prospective	Epilepsy (9.3 ± 3.1), controls (9.5 ± 4)	Epilepsy 20 (38.5%), healthy controls 14 (38.9%)	Epilepsy (17.3 ± 2.5), controls (17.3 ± 1.3)	Generalized 19 (36.5%), focal 33 (63.5%)	NR	NR^c^	Monotherapy 42 (80.8%), polytherapy 10 (19.2%)
e‐Ref #13, 2013	60 (30 epilepsy, 30 healthy controls)	Pediatric and adult	Case‐control study, prospective	Epilepsy (27.60 ± 9.64), controls (29.74 ± 6.89)	Epilepsy 14 (46.7%), healthy controls 14 (46.7%)	Epilepsy (23.43 ± 1.53), controls (23.13 ± 1.10)	Generalized 30 (100%)	Newly diagnosed	28 (93.3%)	Untreated
e‐Ref #15, 2018	120 (60 epilepsy, 60 healthy controls)	Pediatric	Case‐control study, prospective	Epilepsy (11.3 ± 3.1), controls (12.1 ± 2.9)	Epilepsy 27 (45.0%), healthy controls 26 (43.3%)	NR, within normal limit in both groups	Generalized 10 (16.7%), focal 40 (66.7%)	2.03 ± 0.96	NR	Monotherapy 60 (100%)
e‐Ref #20, 2019	70 (30 epilepsy, 40 healthy controls)	Pediatric	Case‐control study, prospective	Epilepsy (10.4 ± 2.9), controls (10.8 ± 3.2)	Epilepsy 18 (60.0%), healthy controls 18 (45.0%)	Epilepsy (17.1 ± 2.6), controls (17.2 ± 3.4)	NR	Newly diagnosed	NR	NR
e‐Ref #30, 2020	129 (66 epilepsy, 63 healthy controls)	Adult	Case‐control study, prospective	Epilepsy (29.4 ± 12.2), controls (33.3 ± 12.1)	NR	Epilepsy (30.1 ± 3.9), controls (28.5 ± 4.7)	NR	12.5 ± 4.5	66 (100%)	Monotherapy 66 (100%)
e‐Ref #31, 2018	91 (46 epilepsy, 45 healthy controls)	Pediatric and adult	Case‐control study, prospective	Epilepsy (25.3 ± 6.2), controls (26.7 ± 5.1)	Epilepsy 32 (69.56%), healthy controls 30 (66.66%)	Epilepsy (24.2 ± 5.0), controls (23.9 ± 5.3)	Generalized 46 (100%)	10.43 ± 6.67	NR	Monotherapy 38 (82.6%), polytherapy 8 (17.4%)
e‐Ref #37, 2021	80 (40 epilepsy, 40 healthy controls)	Pediatric	Case‐control study, prospective	Epilepsy (8.95 ± 2.89), controls (7.90 ± 2.84)	Epilepsy 28 (70.0%), healthy controls 24 (60.0%)	NR	Generalized 24 (60.0%), focal 16 (40.0%)	4.42 ± 2.79	NR	NR

Abbreviations: BMI, body mass index; NR, not reported.

^a^ Data presented as median (range).

^b^ Tonic‐clonic seizures occurred in the week prior to study enrolment.

^c^ Inclusion criteria consisted of no tonic‐clonic seizures during the 12 mo prior to study enrolment.

**FIGURE 2:**
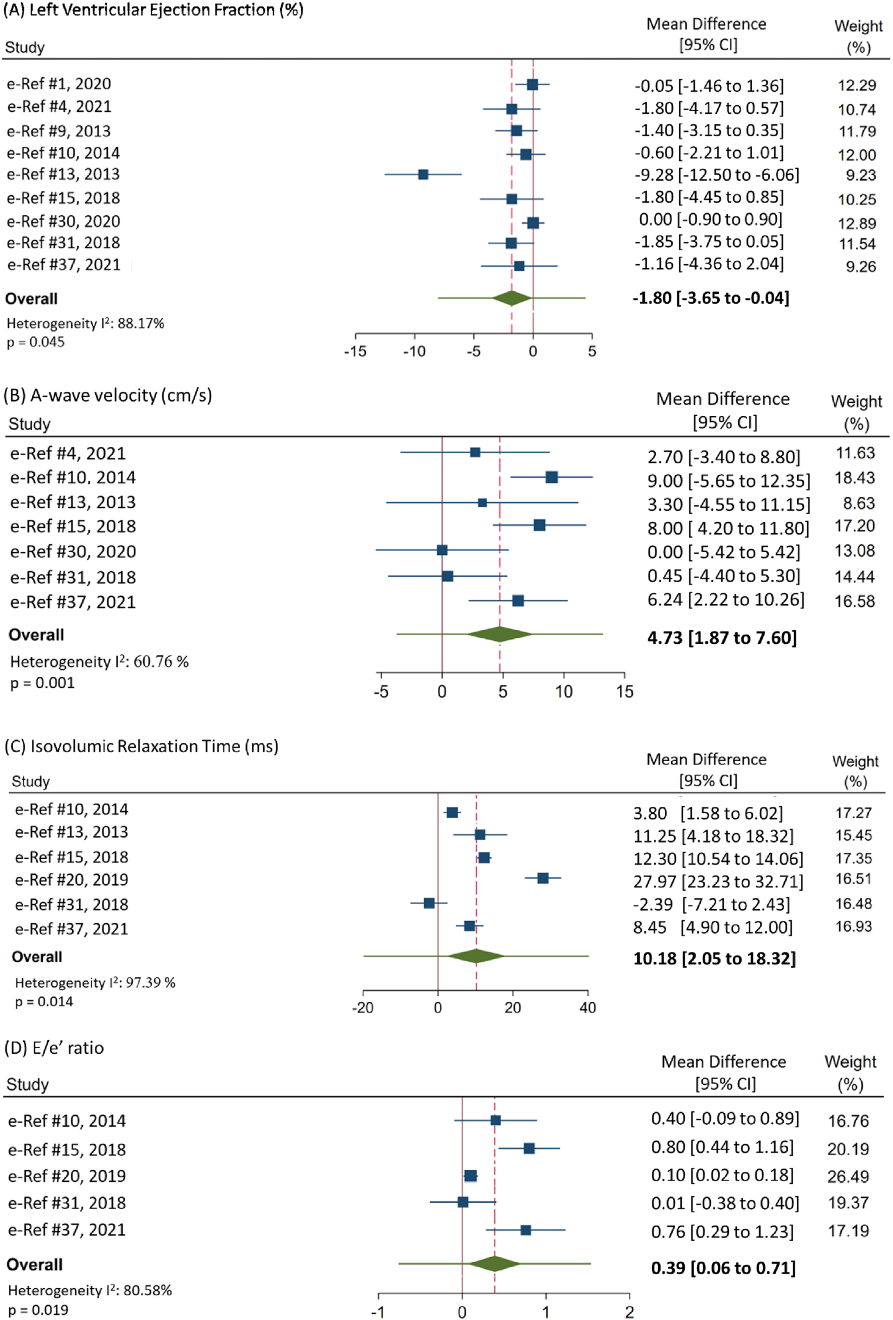
Forest plots of mean difference for each meta‐analysis. Forest plots for meta‐analyses for LVEF (A), A‐wave velocity (B), IVRT (C), E/e' ratio (D). The green diamond represents the overall 95% confidence interval, and the green bar represents the overall 95% prediction interval. 95% prediction interval: LVEF: (−7.99 to 4.39), A‐wave velocity: (−3.71 to 13.17), IVRT: (−19.70 to 40.06), E/e': (−0.75 to 1.53).

Compared with healthy controls, patients with epilepsy had decreased LVEF (MD: −1.80; 95% CI: −3.56 to −0.04; *P* = 0.045; *I* ^2^ = 88.17%), greater late filling A‐wave velocity (MD: 4.73; 95% CI: 1.87‐7.60; *P* = 0.001; *I* ^2^ = 60.76%), prolonged IVRT (MD: 10.18; 95% CI: 2.05‐18.32; *P* = 0.014; *I* ^2^ = 97.39%), and increased E/e' ratio (MD: 0.39; 95% CI: 0.06‐0.71; *P* = 0.019; *I* ^2^ = 81.06%; Figure 2). There were no differences in FS, *E* wave velocity, E/A ratio, *E* wave deceleration time, or e' wave velocity between people with epilepsy and healthy controls (Table S7). There were no differences found in any structural echocardiogram parameters (Table S7).

#### 3.4.2 Meta‐regression analysis

Results of the meta‐regression analysis are shown in Table S8. Sex, BMI, the percentage of patients with generalized epilepsy, and the percentage of patients with monotherapy were not the major source of heterogeneity and were not associated to any of the echocardiography differences between epilepsy and healthy participants. Age was significantly associated with and could explain a substantial amount of heterogeneity in A‐wave velocity (study number = 7; *P* = 0.010; *R* ^2^ = 64.07%) and FS (study number = 6; *P* = 0.035; *R* ^2^ = 45.17%), while it was not associated with LVEF, IVRT, or E/e' ratio (Table S8). Year since epilepsy diagnosed in patients contributed to a substantial amount of heterogeneity in LVEDD (study number = 5; *P* = 0.290; *R* ^2^ = 43.33%), LVESD (study number = 5; *P* = 0.041; *R* ^2^ = 49.61%), and interventricular septum diameter at end‐diastole (study number = 5; *P* = 0.053; *R* ^2^ = 47.91%).

### 3.5 Descriptive analysis

#### 3.5.1 Studies assessing the effect of epilepsy treatments on echocardiogram findings

Seven studies reported echocardiography results before and after treatment, including valproate therapy (n = 1), carbamazepine therapy (n = 1), ketogenic diet (KD; n = 3), adrenocorticotropic hormone (ACTH) therapy (n = 1), and vagus nerve stimulation (VNS; n = 1). These studies are summarized in Table S9. In studies investigating the effect of the ASMs valproic acid or carbamazepine, there was no evidence of echocardiogram abnormalities with prolonged (>6 months) use.^28^, ^29^ There was no difference in echocardiogram findings before and 12 months following VNS implantation in one study.^30^ In a case‐control study that investigated the effect of the KD in young adults with epilepsy, echocardiogram findings did not differ between KD treated and control groups, but parameters of arterial stiffness were increased in the treated group (>6 months treatment).^31^ In another study, KD (6 months) was associated with decreased e' velocity and e'/a' ratio, which is considered to be related to right ventricular diastolic dysfunction.^32^ However, there was no change in echocardiography parameters after 13 months of KD in another study.^33^ ACTH therapy was associated with increased the LV mass index, which resolved following treatment discontinuation.^34^

#### 3.5.2 Other studies

There were two studies that reported cardiac echocardiogram findings in patients with SE. In one study, reduced LVEF was observed in 18/32 patients 6 h following ICU admission.^10^ In another study, elevated plasma cardiac Troponin I was observed in 33 patients with convulsive SE, with a significant increase in LVEDD and LVESD demonstrated in patients compared with healthy controls.^11^ Two studies reported normal echocardiography results in patients with either complicated (epilepsy with systemic complications) or uncomplicated epilepsy; however, no comparisons were made.^22^, ^27^

### 3.6 Postmortem findings

#### 3.6.1 Studies assessing SUDEP

Postmortem cardiac findings including cardiac structure and heart weight were reported in 16 studies (Table 2). In the six studies that examined cardiovascular comorbidities, there were no differences in the frequency of comorbid hypertension, diabetes, or hyperlipidemia between epilepsy patients and controls. Ten studies examined SUDEP population. Pooled analysis of cardiac hypertrophy, heart weight, cardiac interstitial, or perivascular fibrosis in SUDEP cases (n = 714) were performed in nine studies that were rated of “moderate” to “good” quality (Table 3). The pooled prevalence of cardiac hypertrophy from five studies was 16% (95% CI: 9%‐23%), and cardiac fibrosis from seven studies was 20% (95% CI: 15%‐26%). There were no differences reported in cardiac hypertrophy, heart weight, or cardiac fibrosis between SUDEP cases and epilepsy controls except in one study: the prevalence of cardiac hypertrophy was increased in epilepsy deaths that were not related to SUDEP (21.6%) compared with SUDEP cases (13.7%).^35^ In another study, the prevalence of cardiac fibrosis was increased in SUDEP cases (40%) compared with noncoronary sudden deaths among people without epilepsy (6.6%).^17^ In the study rated as “poor” quality that was not included in the pooled analysis, the heart weight and the incidence of cardiac hypertrophy and scar tissue were significantly higher in people with epilepsy who died from cardiovascular causes compared with the SUDEP group.^24^

**TABLE 2:** Characteristics of studies included in the systematic review of postmortem cardiac findings.

Study	Population size (n)	Population	Study design	Age (y), mean ± SD	BMI (kg/m^2^), mean ± SD	Female sex, n (%)	SUDEP classification
e‐Ref #2, 2020	1086 SUDEP	Adult	Cohort study, retrospective	SUDEP 39 (0.25‐81)^a^	NR	400 (36.8%)	Definite/probable/near SUDEP or SUDEP plus
e‐Ref #3, 2020	104 (46 SUDEP, 58 non‐SUDEP)	Adult	Cohort study, retrospective	SUDEP 32 (19‐45), non‐SUDEP 35 (19‐45)^a^	NR	SUDEP 17 (37.0%), non‐SUDEP 19 (32.8%)	Definite or probable SUDEP
e‐Ref #8, 2012	74 SUDEP	Pediatric and Adult	Case‐series study, retrospective	SUDEP (16‐43)^b^	NR	31 (41.9%)	NR
e‐Ref #11, 2013	2306 (106 SCA with epilepsy, 2311 SCA without epilepsy)	Adult	Cohort study, retrospective	SCA with epilepsy (55 ± 25), SCA without epilepsy (63 ± 19)	NR	SCA with epilepsy (42%), SCA without epilepsy (33%)^c^	NA
e‐Ref #12, 2015	10 745 (912 SCD, 9832 non‐SCD	NR	Case‐control study, retrospective	SCD (71.7 ± 13.7), non‐SCD (66.1 ± 13.6)	NR	SCD 352 (38.0%), non‐SCD 3513 (35.7%)	NA
e‐Ref #14, 2005	33 (11 SE, 22 healthy controls)	Pediatric and Adult	Case‐series study, retrospective	SE (50.27 ± 32.19), healthy controls (65.55 ± 19.54)	NR	SE 3 (27.3%), healthy controls 10 (45.5%)	NA
e‐Ref #16, 2004	133 (57 SUDEP, 76 non‐SUDEP)	Pediatric and Adult	Case‐control study, retrospective	Overall 44 (14‐77)^d^	NR	NR	NR
e‐Ref #17, 2017	54 (40 SUDEP, 14 non‐SUDEP)	NR	Cohort study, retrospective	SUDEP (30.6 ± 13.3), non‐SUDEP (44.4 ± 13.1)	NR	38 (33.9%)^e^	NR
e‐Ref #18, 1976	9 epilepsy	Adult	Case series study, retrospective	Epilepsy (23‐44)^b^	NR	4 (44.4%)	NA
e‐Ref #21, 1991	7 SUDEP	NR	Case series study, retrospective	NR	NR	NR	NR
e‐Ref #22, 2018	159 (12 SUDEP, 90 SAD, 57 non‐epilepsy trauma death)	NR	Cross‐sectional study, prospective	SUDEP (55.17 ± 17.23), SAD (61.73 ± 14.68), Trauma (51.39 ± 18.65)	SUDEP (26.17 ± 3.03), SAD (30.72 ± 9.72), Trauma (27.22 ± 5.05)	SUDEP 4 (33.3%), SAD 16 (17.8%), non‐epilepsy trauma 15 (26.3%)	Definite or possible SUDEP
e‐Ref #23, 1986	53 (23 epilepsy, 30 healthy controls)	Adult	Case‐control study, retrospective	Epilepsy (32), healthy controls (43)	NR	Epilepsy 12 (52.2%), healthy controls 16 (53.3%)	NA
e‐Ref #24, 1998	37 (7 epilepsy, 13 healthy controls)	Pediatric and adult	Case‐control study, retrospective	Epilepsy (12‐44), healthy controls (18‐37)^b^	NR	Epilepsy 2 (28.6%), healthy controls 4 (30.8%)	NA
e‐Ref #25, 2005	30 (15 SUDEP, 15 non‐epilepsy noncoronary sudden death)	Adult	Case‐control study, retrospective	SUDEP (40 ± 13), controls (38 ± 7)	NR	SUDEP 9 (60.0%)^e^	NR
e‐Ref #33, 1991	44 SUDEP	Pediatric and adult	Case series study, retrospective	SUDEP (3‐58)^a^	NR	16 (36.4%)	NR
e‐Ref #36, 2021	154 (96 SUDEP, 58 non‐SUDEP)	Pediatric and adult	Cohort study, retrospective	SUDEP (36.9 ± 19.6), non‐SUDEP (53.0 ± 18.3)	SUDEP (26.5 ± 8.4), non‐SUDEP (23.1 ± 8.1)	SUDEP 40 (42.5%), non‐SUDEP 18 (31.0%)	Definite/definite‐plus, probable/or possible SUDEP

Abbreviations: BMI, body mass index; NR, not reported; SAD, sudden arrhythmic death; SCA, sudden cardiac arrest; SCD, sudden cardiac death; SUDEP, sudden unexpected death in epilepsy; non‐SUDEP, non‐SUDEP death in epilepsy.

^a^ Indicates median (range).

^b^ Indicates range.

^c^ Number of patients not reported.

^d^ Indicates mean (range).

^e^ Sex of individual subgroups not reported.

**TABLE 3:** Postmortem cardiac findings in SUDEP cases

Study	Autopsied cases (n)	Age (y), mean ± SD	BMI (kg/m^2^), mean ± SD	Female sex, n (%)	Heart weight (g), mean ± SD	Cardiac hypertrophy, n (%)	Cardiac interstitial or perivascular fibrosis, n (%)
e‐Ref #2, 2020	570 (380 SUDEP, 190 non‐SUDEP)	SUDEP 42 (18‐67), non‐SUDEP 42 (18‐67)^a^	SUDEP 27.8 (12.4‐69.2), non‐SUDEP 26.3 (16.9‐58)^a^	SUDEP 144 (37.9%), non‐SUDEP 72 (37.9%)	No difference	SUDEP 52 (13.7%), non‐SUDEP 41 (21.6%)^d^	SUDEP 73 (19.2%), non‐SUDEP 39 (20.5%)
e‐Ref #3, 2020	83 (42 SUDEP, 41 non‐SUDEP)	NR	NR	NR	SUDEP 380 (150‐640), non‐SUDEP 370 (150‐520)^a^	SUDEP 14 (33.3%), non‐SUDEP 13 (31.7%)	SUDEP 11(26.2%), non‐SUDEP 6 (14.6%)
e‐Ref #8, 2012	74 SUDEP	SUDEP (14‐63)^a^	NR	31 (41.9%)	SUDEP 378 (230‐520)^a^	SUDEP 7 (9.5%)	SUDEP 22 (42.3%)
e‐Ref #16, 2004	65 (57 SUDEP, 8 non‐SUDEP)	NR	NR	NR	SUDEP 358, non‐SUDEP 399	NR	NR
e‐Ref #21, 1991	7 SUDEP	NR	NR	NR	NR	SUDEP 1 (14.3%)	SUDEP 1 (14.3%)
e‐Ref #22, 2018	159 (12 SUDEP, 90 SAD, 57 trauma death)	SUDEP (55.17 ± 17.23), SAD (61.73 ± 14.68), Trauma (51.39 ± 18.65)	SUDEP (26.17 ± 3.03), SAD (30.72 ± 9.72), Trauma (27.22 ± 5.05)	SUDEP 4 (33.3%), SAD 16 (17.8%), Trauma 15 (26.3%)	SUDEP (446.67 ± 155.41), SAD (571.61 ± 158.20), Trauma (391.93 ± 108.85)	NR	SUDEP cases had less fibrosis than SAD cases.
e‐Ref #25, 2005	30 (15 SUDEP, 15 non‐epilepsy noncoronary sudden death)	SUDEP (40 ± 13), controls (38 ± 7)	NR	SUDEP 9 (60.0%)^c^	SUDEP (340 ± 76.2), controls (369 ± 65.2)	NR	Patients 6 (40%), controls 1 (6.6%)^d^
e‐Ref #33, 1991	44 SUDEP	SUDEP (3‐58)^a^	NR	16 (36.4%)	NR	NR	SUDEP 5 (11.4%)
e‐Ref #36, 2021	83 SUDEP	NR	NR	NR	SUDEP (358 ± 138)	SUDEP 14 (16.9%)	SUDEP 14 (34.1%)

Abbreviations: BMI, body mass index; NR, not reported; SAD, sudden arrhythmic death; SUDEP, sudden unexpected death in epilepsy; non‐SUDEP, non‐SUDEP death in epilepsy.

^a^ Indicates median (range).

^b^ Indicates range.

^c^ Sex of individual subgroups not reported.

^d^ Statistically significant finding between patients with epilepsy and controls.

#### 3.6.2 Studies assessing non‐SUDEP deaths

One study reported that epilepsy and use of carbamazepine and gabapentin were associated with increased SCD risk,^36^ while another study found no relationship between seizure and sudden cardiac arrest.^23^ One study found a higher frequency of myocardial contraction bands in patients who died directly from SE compared with those who had a history of SE, but did not die as a direct result of SE.^37^ Two studies examining patients with epilepsy who died from other causes suggested that cardiac pathological conditions may be related to death in epilepsy,^26^, ^38^ while another study found no structural differences in myocardium between epilepsy and nonepilepsy controls.^25^

## 4 DISCUSSION

This systematic review of 37 studies on cardiac structure and function in epilepsy reveals several clinically valuable findings. Our primary analysis found that several systolic and diastolic echocardiography parameters were altered in a relatively young epilepsy population without any underlying cardiovascular disease. Of importance, we found decreased LVEF in patients with epilepsy compared with controls, which is the “gold‐standard” marker for systolic dysfunction and is associated with substantial cardiovascular morbidity and mortality in cardiovascular disease populations. In contrast, SUDEP was not associated with an increased prevalence of cardiac structural abnormalities, including cardiac hypertrophy, heart weight, or cardiac fibrosis, compared with epilepsy controls.

Compared with healthy controls, individuals with epilepsy had decreased LVEF. LVEF is an important marker in assessing LV function and a major diagnostic and prognostic measure for several cardiovascular disease states (heart failure, myocardial infarction, and valvular heart disease).^39^ Importantly, LVEF values for epilepsy across studies were within a clinically normal range (62.8%‐70.9%), but given that these studies consisted of young and otherwise healthy participants without underlying cardiovascular disease, the significance and mechanisms underlying this are important to understand. Enhanced sympathetic stimulation in epilepsy may result in catecholamine toxicity of the myocardium.^6^ Excessive catecholamine release can cause acute but reversible cardiomyopathy including apical ballooning, LV dysfunction, and ST‐segment elevation, as well as decreased LVEF.^40^ In addition, stress‐related cardiomyopathy is common in patients with convulsive SE.^10^

We also found several other measures associated with diastolic dysfunction including increased A‐wave velocity, E/e' ratio, and IVRT in people with epilepsy. The elevation of peak A‐wave velocity may underlie LV stiffness and LA contractility changes. Increased cardiac stiffness may potentially result from excessive extracellular matrix deposition in myocardial fibrosis, which can lead to myocardial electrophysiological remodeling and arrhythmias.^41^ In our review, patients with epilepsy had a higher E/e' ratio compared with healthy controls, which is associated with increased LV filling pressure and serves as an independent predictor of primary cardiac events in hypertensive patients.^42^ Increased IVRT reflects the time latency between the onset of aortic valve closure to the onset of mitral valve opening to allow for ventricular filling, and it is strongly associated with hypertension.^43^ The difference in A‐wave velocity found between patients and healthy participants was negatively associated with age, suggesting that the diastolic function in younger patients may be more easily affected by epilepsy.

Importantly, participants in our meta‐analysis were free of cardiovascular disease including chronic hypertension. Impaired baroreflex sensitivity and catecholamine toxicity during the postictal period following tonic‐clonic seizures may contribute toward the dysregulation of blood pressure,^44^ leading to subsequent intermittent hypertension and changes in diastolic function. Transient changes to hypertension are common with seizures, particularly tonic‐clonic seizures^45^; it is possible that repetitive seizures may lead to increased arterial stiffness and modulation of systemic blood pressure over time. In patients with hypertension, the abnormal response to hemodynamical challenge is related to impaired diastolic filling, which is caused by decreased left ventricular relaxation and compliance.^46^ Tonic‐clonic seizures have been associated with increased left atrial diameter.^47^ Increased sympathetic tone in epilepsy as a result of recurrent seizures may make cardiac arrhythmias and arterial blood dysregulation more likely.^44^ Indeed, enhanced sympathetic tone is also a marker of elevated SCD risk.^48^ It has also been suggested that seizures may be associated with cardiac ischemia, which can potentially undermine diastolic function.^49^ Other cardiovascular disease risk factors such as age or obesity were unlikely to contribute toward this difference, where included participants were young (mean age range, 9.3‐37.4 years), and there were no differences in BMI between epilepsy patients and controls. Patients with epilepsy are less physically active and more likely to adopt a sedentary lifestyle than healthy population,^50^, ^51^ which may also increase their risk of impaired cardiac output and cardiac stiffness.^52^

In a recently published systematic review, lamotrigine was not associated with sudden death and ECG abnormalities in epilepsy.^53^ In our systematic review, similarly, there was no evidence showing that long‐term use of valproic acid, carbamazepine, VNS, or ACTH therapy was associated with echocardiographic abnormalities in studies that evaluated the effect of epilepsy therapies on cardiac structure and function. However, the KD was reported to influence cardiac structure and systolic/diastolic function. In a recent animal model study, the KD inhibited transcription of mitochondrial ribosome‐encoding genes, which was associated with an increased risk of atrial fibrillation and cardiac fibrosis.^54^

We found that SUDEP was not associated with significant structural cardiac abnormalities, compared with non‐SUDEP controls with epilepsy. This is consistent with previous investigations suggesting that cardiac dysfunction may not be a primary mechanism of death in these cases, which is instead driven by centrally mediated cardiorespiratory terminal arrest.^55^ However, there are caveats relating to SUDEP epidemiology where there is a strong overlap between SCD and SUDEP, and many cases of SUDEP may be mistaken for a sudden death of a primary cardiac cause.^56^, ^57^ In addition, early‐stage cardiomyopathies may only demonstrate mild microscopic and macroscopic signs, making the forensic diagnosis challenging in some cases.^58^ Pathogenic genetic variants encoding cardiac disease (eg, cardiac channelopathies) may also contribute toward arrhythmic death in people without structural disease.^59^ Molecular autopsy (postmortem genetic analysis) of cardiomyopathies‐ and channelopathies‐related genes may identify such cases and should be considered when addressing the cause of death in epilepsy population.^58^ An evaluation of the genetic variants expressed in the brain found that SUDEP patients had multiple variants associated with cardiac arrhythmia, which was not observed in living epilepsy controls.^60^

Our systematic review and meta‐analysis explored major structural and functional cardiac changes in the epilepsy population. However, there are also limitations. Studies were excluded if they used indirect measurements of heart structure alone (eg, electrocardiography). Several studies were also excluded during the full‐text review as they did not report quantitative results in echocardiography and postmortem findings. Further, to minimize reporting bias, we only included studies that were rated as “fair” to “good” quality in our meta‐analysis and pooled analysis. We were unable to study disease progression as most echocardiogram studies were conducted at one time point. Due to the small sample size, we were unable to examine the role of potential epilepsy confounders such seizure frequency, drug resistant epilepsy, history of SE, history of tonic‐clonic seizures, or specific medication use as these were not comprehensively reported most studies. Additionally, the lack of individual patients' data prevented further investigation in specific cardiac risk factors for our meta‐regression, including genetic etiology that could contribute to both epilepsy and heart disease. Although we excluded echocardiogram studies of patients with preexisting cardiovascular disease, the heterogeneity of exclusion criteria applied across studies may have impacted on our findings. The heterogeneity of effect size in echocardiography was high and was only partly explained by the moderators explored. The heterogeneity caused by age or epilepsy duration could not be further explored in subgroup comparisons due to the small sample size. Our investigations were performed using across different modalities and technicians, which may also lead to interstudy differences. We were also unable to include multiple studies that presented descriptive but not numerical echocardiography findings.

## 5 CONCLUSION

This systematic review and meta‐analysis addresses a gap in the literature regarding characterizing the specific changes to cardiac structure and function that occur in people with epilepsy and further supports the concept of the “epileptic heart.”^2^ We found several echocardiogram parameters that were altered in patients with epilepsy, which may collectively indicate early markers of LV diastolic and systolic function. While the prognostic significance of these cardiac changes remains unclear, these changes may shed light on the underlying mechanisms, which may lead to preventative strategies. Longitudinal studies are required to understand disease progression and the prognostic significance of such changes in epilepsy, including whether these changes are associated with an increased risk of cardiovascular‐related morbidity and mortality. Echocardiography may potentially be an important cardiac diagnostic tool in epilepsy but will be informed by prospective studies to identify “at‐risk” patients to optimize their clinical management.

## AUTHOR CONTRIBUTIONS

Conception and design and drafting the article: Liu and Sivathamboo. Acquisition of data: Liu, Thergarajan, Antonic‐Baker, Chen, Sparks, Lannin, Perucca, O'Brien, and Sivathamboo. Analysis and interpretation of data: Liu, Thergarajan, Chen, and Sivathamboo. Statistical analysis: Chen. Critically revising the article: Liu, Thergarajan, Antonic‐Baker, Chen, Sparks, Lannin, Kwan, Jones, Casillas‐Espinosa, Perucca, O'Brien, and Sivathamboo. Reviewed submitted version of manuscript: All authors. Supervision: O'Brien and Sivathamboo.

## FUNDING INFORMATION

The Australian National Health and Medical Research Council (APP1091593) and the Victorian Medical Research Acceleration Fund.

## CONFLICT OF INTEREST

Z. Liu, P. Thergarajan, A. Antonic‐Baker, P.B. Sparks, and N.C. Jones reports no relevant disclosures relevant to the manuscript. Z. Chen is supported by an Early Career Fellowship from the National Health and Medical Research Council (NHMRC) of Australia (GNT1156444), and his institution has received consultancy fees and/or research grants from NHMRC, Arvelle Therapeutics, and UCB Pharma outside the submitted work. N. A. Lannin is supported by fellowship from the National Heart Foundation (GNT102005), she reports no further relevant disclosures. P. Kwan is supported by a Medical Research Future Fund Practitioner Fellowship (MRF1136427). He reports grants to his institution from Chiesi, Eisai, UCB Pharma, and LivaNova, outside the submitted work. P. M. Casillas‐Espinosa is supported by an Early Career Fellowship from the National Health and Medical Research Council (APP1087172) and The Brain Foundation of Australia. He has received research grants from Supernus Pharmaceuticals, Praxis, Eisai, and Kaoskey outside the submitted work. P. Perucca is supported by an Early Career Fellowship from the National Health and Medical Research Council (APP1163708), the Epilepsy Foundation, the Royal Australasian College of Physicians, The University of Melbourne, Monash University, the Weary Dunlop Medical Research Foundation, Brain Australia, and the Norman Beischer Medical Research Foundation. He has received speaker honoraria or consultancy fees to his institution from Chiesi, Eisai, LivaNova, Novartis, Sun Pharma, Supernus, and UCB Pharma, outside the submitted work. He is an Associate Editor for Epilepsia Open. T. J. O'Brien is supported by a Program Grant (APP1091593) and Investigator Grant (APP1176426) from the National Health and Medical Research Council of Australia and the Victorian Medical Research Acceleration Fund. He reports grants and consulting fees paid to his institution from Eisai, UCB Pharma, Praxis, Biogen, ES Therapeutics, and Zynerba. S. Sivathamboo is supported by a Bridging Postdoctoral Fellowship from Monash University (BPF20‐3253672466) and the Victorian Medical Research Acceleration Fund. She reports salary support from Kaoskey and Optalert for clinical trial related activities; she receives no personal income for these activities. Statistical analysis performed by Dr. Zhibin Chen (PhD, CStat) of Monash University. We confirm that we have read the Journal's position on issues involved in ethical publication and affirm that this report is consistent with those guidelines.

## Supporting information


Appendix S1.

## Data Availability

The full dataset and statistical analysis codes will be made available on reasonable request from any qualified researcher.
