# Supplementary material for: Cardiac structural and functional abnormalities in epilepsy: A systematic review and meta‐analysis
Source: Epilepsia Open. 2023 Jan 31;8(1):46–59. doi: 10.1002/epi4.12692 (PMC9977759; doi:10.1002/epi4.12692)
Supplement: Supplementary file 1 — Appendix S1. [file EPI4-8-46-s001.docx]

**eAppendix 1 Search strategy.**

1. Search string with Boolean operators

**
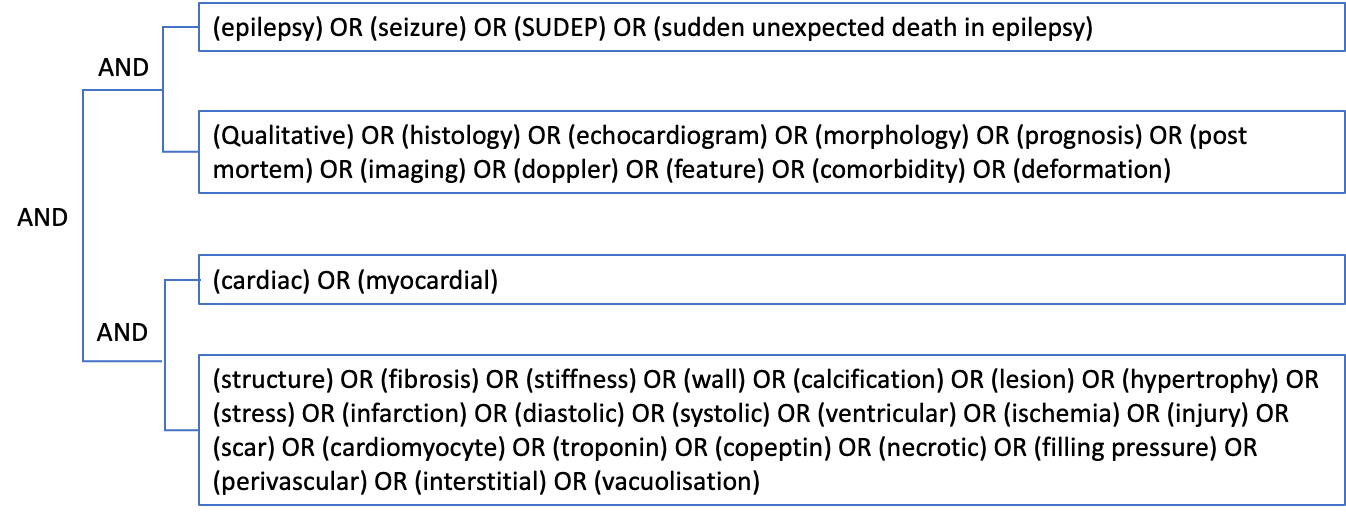
**

1. MEDLINE
   1. (Epilepsy or seizure or SUDEP or sudden unexpected death in epilepsy).af.
   2. (Qualitative or histology or echocardiogram or morphology or post mortem or imaging or doppler or feature or deformation or prognosis or comorbidity).af.
   3. (Cardiac or myocardial).af.
   4. (Structure or fibrosis or stiffness or wall or calcification or hypertrophy or stress or diastolic or systolic or ventricular or ischemia or injury or scar or cardiomyocyte or troponin or copeptin or necrotic or filling pressure or perivascular or interstitial or vacuolization or lesion or infarction).af.
   5. 1 and 2
   6. 3 and 4
   7. 5 and 6
2. PubMED
   1. (((Cardiac) OR (myocardial)) Filters: Humans, English
   2. ((((((((((((((((((((((Structure) OR (fibrosis)) OR (stiffness)) OR (wall)) OR (calcification)) OR (lesion)) OR (hypertrophy)) OR (stress)) OR (infarction)) OR (diastolic)) OR (systolic)) OR (ventricular)) OR (ischemia)) OR (injury)) OR (scar)) OR (cardiomyocyte)) OR (troponin)) OR (copeptin)) OR (necrotic)) OR (filling pressure)) OR (perivascular)) OR (interstitial)) OR (vacuolisation) Filters: Humans, English
   3. (((epilepsy) OR (seizure)) OR (SUDEP)) OR (sudden unexpected death in epilepsy) Filters: Humans, English
   4. ((((((((((Qualitative) OR (histology)) OR (echocardiogram)) OR (morphology)) OR (prognosis)) OR (post mortem)) OR (imaging)) OR (doppler)) OR (feature)) OR (comorbidity)) OR (deformation) Filters: Humans, English
   5. 1 and 2
   6. 3 and 4
   7. 5 and 6
3. COCHRANE
   1. (Epilepsy) OR (Seizure) OR (SUDEP) OR (sudden unexpected death of epilepsy) (Word variations have been searched)
   2. (Qualitative) OR (histology) OR (echocardiogram) OR (morphology) OR (prognosis) OR (imaging) OR (post mortem) OR (doppler) OR (feature) OR (comorbidity) OR (deformation) (Word variations have been searched)
   3. (cardiac) OR (myocardial) (Word variations have been searched)
   4. (structure) OR (fibrosis) OR (stiffness) OR (wall) OR (calcification) OR (lesion) OR (hypertrophy) OR (stress) OR (infarction) OR (diastolic) OR (systolic) OR (ventricular) OR (ischemia) OR (injury) OR (scar) OR (cardiomyocyte) OR (troponin) OR (copeptin) OR (necrotic) OR (filling pressure) OR (perivascular) OR (interstitial) OR (vacuolisation) (Word variations have been searched)
   5. 1 and 2
   6. 3 and 4
   7. 5 and 6
4. Web of Science
   1. TOPIC: (epilepsy) OR TOPIC: (seizure) OR TOPIC: (sudep) OR TOPIC: (sudden unexpected death in epilepsy)
   2. TOPIC: (Qualitative) OR TOPIC: (histology) OR TOPIC: (echocardiogram) OR TOPIC: (morphology) OR TOPIC: (prognosis) OR TOPIC: (post mortem) OR TOPIC: (imaging) OR TOPIC: (doppler) OR TOPIC: (feature) OR TOPIC: (comorbidity) OR TOPIC: (deformation)
   3. TOPIC: (cardiac) OR TOPIC: (myocardial) OR TOPIC: (heart) OR TOPIC: (cardiovascular)
   4. TOPIC: (structure) OR TOPIC: (fibrosis) OR TOPIC: (stiffness) OR TOPIC: (wall) OR TOPIC: (calcification) OR TOPIC: (lesion) OR TOPIC: (hypertrophy) OR TOPIC: (stress) OR TOPIC: (infarction) OR TOPIC: (diastolic) OR TOPIC: (systolic) OR TOPIC: (ventricular) OR TOPIC: (ischemia) OR TOPIC: (injury) OR TOPIC: (scar) OR TOPIC: (cardiomyocyte) OR TOPIC: (troponin) OR TOPIC: (copeptin) OR TOPIC: (necrotic) OR TOPIC: (filling pressure) OR TOPIC: (perivascular) OR TOPIC: (interstitial) OR TOPIC: (vacuolisation)
   5. 1 and 2
   6. 3 and 4
   7. 5 and 6

**Supplementary Table 1: Summary of variables extracted.**

| **Patients Demographics** | **Echocardiography Parameters** |
| --- | --- |
| Population (n) | Left ventricle ejection fraction (%) |
| Age (years) | The peak velocities of early (E) mitral wave velocity (cm/s) |
| Sex (%Female) | The peak velocities of late (A) mitral wave velocity (cm/s) |
| Body Mass Index (kg/m^2^) | E/A ratio |
|  | Lateral peak early diastolic annular (e’) wave velocity (cm/s) |
| **Epilepsy Characteristics** | E/e’ ratio |
| Epilepsy Classification | Isovolumic relaxation time (ms) |
| Generalised (n) | E-wave deceleration time (ms) |
| Focal (n) | Fractional shortening (%) |
| Combined (n) | Left ventricular mass index (mg/m^2^) |
| Unknown (n) | Left ventricular end-diastolic diameter (cm) |
| Newly-diagnosed (n) | Left ventricular end-systolic diameter (cm) |
| Drug-resistant/refractroy (n) | Posterior wall thickness at end-diastole (cm) |
| Year since diagnosed (years) | Interventricular septum diameter at end-diastole (cm) |
| History of tonic-clonic seizures (n) | Left atrium diameter (cm) |
| History of status epilepticus (n) | Left ventricular end-diastolic volume (mL) |
| Mono ASM Treatment (n) |  |
| Poly ASM Treatment (n) | **Post-mortem Parameters** |
|  | Heart weight (g) |
| **Cardiac Disease Characteristics** | Cardiac fibrosis (n) |
| Cardiac and respiratory comorbidities (included (n), excluded, not reported) | Cardiac hypertrophy (n) |
| Exclusion criteria for cardiac disease |  |
| Cardiovascular treatment (drug, number) |  |

**Supplementary Table 2: Summary of studies meeting inclusion and exclusion criteria that underwent study quality appraisal.**

| **Study** | **Population size (n)** | **Age group** | **Age (years), mean ± SD** | **Female sex, n (%)** | **Study design** | **Overall quality rating** | **Cardiac risk factors reported** |
| --- | --- | --- | --- | --- | --- | --- | --- |
| Schreiber et al. 2020^e1^ | 82 (41 epilepsy, 41 healthy controls) | Pediatric | Epilepsy (10.1 ± 4.6), controls (10.1 ± 5.6) | Epilepsy 17 (41.5%), control 17 (41.5%) | Case-control study, prospective | Fair | Participants with history of cardiac disease were excluded |
| Cihan et al. 2020^e2^ | 1086 SUDEP | Adult | SUDEP 39 (0.25-81)^a^ | 400 (36.8%) | Cohort study, retrospective | Good | Diabetes, COPD, hypertension |
| Karlovich et al. 2020^e3^ | 104 (46 SUDEP, 58 non-SUDEP) | Adult | SUDEP 32 (19-45), non-SUDEP 35 (19-45)^a^ | SUDEP 17 (37.0%), non-SUDEP 19 (32.8%) | Cohort study, retrospective | Fair | Hypertension, diabetes |
| Fialho et al. 2021^e4^ | 60 (30 epilepsy, 30 healthy controls) | Adult | Epilepsy (37.4 ± 11.2), controls (35.3 ± 9.3) | Epilepsy 18 (60%), healthy controls 18 (60%) | Case-control Study, prospective | Fair | Participants with cardiovascular diseases including greater than mild hypertension were excluded |
| Eskandarian et al. 2011^e5^ | 60 (30 complicated seizure, 30 uncomplicated seizures) | Pediatric and adult | Complicated seizure (43.4 ± 15.5), uncomplicated seizure (44.7 ± 21.5) | Complicated seizures 14 (46.7%), uncomplicated seizures 14 (46.7%) | Case-control study, prospective | Poor | NR |
| Celik et al. 2018^e6^ | 40 epilepsy | Pediatric | 10.03 ± 3.75 | 23 (57.5%) | Cohort study, prospective | Good | NR |
| Belcour et al. 2015^e7^ | 32 SE | Adult | 50 ± 18 | 11 (34.4%) | Cross-sectional study, prospective | Fair | Participants with chronic heart failure or myocardial infarction were excluded. |
| Zhuo et al. 2012^e8^ | 74 SUDEP | Pediatric and adult | SUDEP (16-43)^b^ | 31 (41.9%) | Cohort study, retrospective | Fair | NR |
| Ramadan et al. 2013^e9^ | 180 (120 epilepsy, 60 healthy controls) | Pediatric and Adult | Epilepsy (25.2 ± 9.3), controls (27.3 ± 7.5) | Epilepsy 44 (36.7%), healthy controls 21 (35.0%) | Case-control study, prospective | Fair | Participants with autonomic dysfunction were excluded |
| Kibar et al. 2013^e10^ | 88 (52 epilepsy, 36 healthy controls) | Pediatric | Epilepsy (9.3 ± 3.1), controls (9.5 ± 4) | Epilepsy 20 (38.5%), healthy controls 14 (38.9%) | Case-control study, prospective | Fair | Participants with cardiac abnormalities, hypertension, sleep apnea, blood chemistry abnormalities were excluded |
| Stecker et al. 2013^e11^ | 2306 (106 SCA with epilepsy, 2311 SCA without epilepsy) | Adult | SCA with epilepsy (55 ± 25), SCA without epilepsy (63 ± 19) | SCA with epilepsy (42%), SCA without epilepsy (33%)^b^ | Cohort study, retrospective | Poor | Stroke, hypertension, diabetes, hyperlipidemia |
| Bardai et al. 2015^e12^ | 10745 (912 SCD, 9832 non-SCD) | NR | SCD (71.7 ± 13.7), non-SCD (66.1 ± 13.6) | SCD 352 (38.0%), non-SCD 3513 (35.7%) | Case-control study, retrospective | Good | NR |
| Bilgi et al. 2014^e13^ | 60 (30 epilepsy, 30 healthy controls) | Pediatric and Adult | Epilepsy (27.60 ± 9.64), controls (29.74 ± 6.89) | Epilepsy 14 (46.7%), healthy controls 14 (46.7%) | Case-control study, prospective | Fair | Participants with hypertension, heart disease, diabetes, hyperlipidemia were excluded |
| Manno et al. 2015^e14^ | 33 (11 SE, 22 healthy controls) | Pediatric and adult | SE (50.27 ± 32.19), healthy controls (65.55 ± 19.54) | SE 3 (27.3%), healthy controls 10 (45.5%) | Case-control study, retrospective | Fair | COPD, atrial septal defect, coronary artery disease, hypertension, ventricular septal defect |
| Çelik et al. 2018^e15^ | 120 (60 epilepsy, 60 healthy controls) | Pediatric | Epilepsy (11.3 ± 3.1), controls (12.1 ± 2.9) | Epilepsy 27 (45.0%), healthy controls 26 (43.3%) | Case-control study, prospective | Fair | Participants with coronary artery disease, hypertension or hypotension, acquired cardiac disease were excluded |
| Davis et al. 2004^e16^ | 133 epilepsy (57 SUDEP, 76 non-SUDEP) | Pediatric and Adult | Overall 44 (14-77)^a^ | NR | Case-control study, retrospective | Fair | NR |
| MELEZ et al. 2017^e17^ | 54 epilepsy (40 SUDEP, 14 epilepsy cardiovascular death) | NR | SUDEP (30.6±13.3), non-SUDEP (44.4±13.1) | 38 (33.9%)^b^ | Cohort study, retrospective | Poor | NR |
| Falconer et al. 1976^e18^ | 9 epilepsy | Adult | Epilepsy (23-44)^a^ | 4 (44.4%) | Case-series study, retrospective | Good | Participants with arteriosclerosis and  arteriosclerotic heart disease was excluded |
| Amrousy et al. 2017^e19^ | 60 SE, 30 healthy controls | Pediatric | SE (9.1 ± 2.3), controls (9.9 ± 1.6) | 12 (35.6%) | Case-control study, prospective | Fair | Participants with sepsis, cardiac, hepatic, renal, endocrinal or muscle disease were excluded |
| Tosun et al. 2018^e20^ | 70 (30 epilepsy, 40 healthy controls) | Pediatric | Epilepsy (10.4 ± 2.9), controls (10.8 ± 3.2) | Epilepsy 18 (60.0%), healthy controls 18 (45.0%) | Case-control study, prospective | Fair | Participants with diseases known to effect autonomic function including hypertension, diabetes, smoking were excluded |
| Dasheiff et al. 1991^e21^ | 7 SUDEP | NR | NR | NR | Case-series study, retrospective | Fair | NR |
| Devinsky et al. 2018^e22^ | 159 (12 SUDEP, 90 SAD, 57 non-epilepsy trauma death) | Pediatric and adult | SUDEP (55.17 ± 17.23), SAD (61.73 ± 14.68), non-epilepsy Trauma (51.39 ± 18.65) | SUDEP 4 (33.3%), SAD 16 (17.8%), non-epilepsy trauma 15 (26.3%) | Cross-sectional study, prospective | Fair | NR |
| Vesterby et al. 1986^e23^ | 53 (23 epilepsy, 30 healthy controls) | Adult | Epilepsy (32), healthy controls (43) | Epilepsy 12 (52.2%), healthy controls 16 (53.3%) | Case-control study, retrospective | Poor | NR |
| Natelson et al. 1998^e24^ | 37 (7 epilepsy, 13 healthy controls) | Pediatric and adult | Epilepsy (12-44), healthy controls (18-37)^a^ | Epilepsy 2 (28.6%), healthy controls 4 (30.8%) | Case-control study, retrospective | Poor | Cases with no history or evidence of heart disease |
| P-Codrea et al. 2005^e25^ | 30 (15 SUDEP, 15 non-epilepsy noncoronary sudden death) | Adult | SUDEP (40 ± 13), controls (38 ± 7) | SUDEP 9 (60.0%)^c^ | Case-control study, retrospective | Fair | NR |
| Arhan et al. 2009^e26^ | 40 epilepsy | Pediatric | 9.5 ± 2.8 | 9 (22.5%) | Cohort study, prospective | Fair | Participants with systemic disease, drug usage for any other disease, family history of coronary artery disease were excluded |
| Hajsadeghi et al. 2009^e27^ | 49 epilepsy | Pediatric and adult | 21.18 ± 8.37 | 15 (30.6%) | Cross-sectional study, prospective | Poor | Participants with history of cardiac problems and intervention were excluded |
| Ozdemir et al. 2016^e28^ | 61 epilepsy | Pediatric | 5.4 ± 3.3 | 29 (47.5%) | Cohort study, prospective | Fair | NR |
| Kutluk et al. 2020^e29^ | 18 epilepsy | Pediatric | 0.675 ± 0.3 | 5 (27.8%) | Cohort study, prospective | Fair | NR |
| Asoglu et al. 2020^e30^ | 129 (66 epilepsy, 63 healthy controls) | Adult | Epilepsy (29.4 ± 12.2), controls (33.3 ± 12.1) | NR | Case-control study, prospective | Fair | Participants with cardiac rhythm disorders,  diabetes, bundle branch block, or known cardiac and psychiatric disease were excluded |
| Genc et al. 2018^e31^ | 91 (46 epilepsy, 45 healthy controls) | Pediatric and adult | Epilepsy (25.3 ± 6.2), controls (26.7 ± 5.1) | Epilepsy 32 (69.56%), healthy controls 30 (66.66%) | Case-control study, prospective | Fair | Participants with coronary artery disease, hypertension, valvular heart disease, diabetes were excluded |
| Coppola et al. 2013^e32^ | 43 epilepsy (23 treated, 20 untreated) | Pediatric and adult | Treatment (11 ± 8), control (10 ± 7) | Treated 12 (52.2%), untreated 11 (55.0%) | Case-control study, prospective | Fair | Participants with heart failure, systemic hypertension, diabetes, thyroid dysfunction were excluded |
| Earnest et al. 1991^e33^ | 44 epilepsy | Pediatric and adult | SUDEP (3-58)^a^ | 16 (36.4%) | Cross-sectional study, retrospective | Fair | NR |
| Doksöz et al. 2015^e34^ | 32 epilepsy | Pediatric | 3.79^a^ | 10 (31.3%) | Cohort study, prospective | Fair | Participants with heart disease, hypertension, diabetes, corticosteroids were excluded |
| Cadeddu et al. 2010^e35^ | 10 epilepsy | Pediatric and adult | 32.4 ± 9.1 | 6 (60%) | Cohort study, prospective | Good | Participants with cardiovascular  congenital or acquired diseases were excluded |
| Chahal et al. 2021^e36^ | 154 epilepsy (96 SUDEP, 58 non-SUDEP) | Pediatric and adult | SUDEP (36.9 ± 19.6), non-SUDEP (53.0 ± 18.3) | SUDEP 40 (42.5%), non-SUDEP 18 (31.0%) | Cohort study, retrospective | Fair | NR |
| Ibrahim et al. 2021^e37^ | 80 (40 epilepsy, 40 healthy controls) | Pediatric | Epilepsy (8.95 ± 2,89), controls (7.90 ± 2.84) | Epilepsy 28 (70.0%), healthy controls 24 (60.0%) | Case-control study, prospective | Fair | Participants with acquired or congenital heart diseases, hypertension, and metabolic or endocrine disorders were excluded |

SAD=sudden arrhythmic death; SCA=sudden cardiac arrest; SCD=sudden cardiac death; SE=status epilepticus; SUDEP=sudden unexpected death in epilepsy; non-SUDEP = Non-SUDEP death in epilepsy; NR=not reported.

^a^Data was reported as Median (Range) or (Range).

^b^Number of patients not reported.

^c^Sex of individual subgroups not reported.

**Supplementary Table 3: Quality appraisals for observational cohort and cross-sectional studies using the NHLBI study quality assessment tool.**

| **Study** | **Q1** | **Q2** | **Q3** | **Q4** | **Q5** | **Q6** | **Q7** | **Q8** | **Q9** | **Q10** | **Q11** | **Q12** | **Q13** | **Q14** | **Overall quality rating** |
| --- | --- | --- | --- | --- | --- | --- | --- | --- | --- | --- | --- | --- | --- | --- | --- |
| e-Ref #2 | Yes | Yes | Yes | Yes | No | No | Not applicable | Yes | Yes | Not applicable | Yes | No | Not applicable | Yes | Good |
| e-Ref #3 | Yes | Yes | Yes | Yes | No | No | Not applicable | No | Yes | Not applicable | Yes | No | Not applicable | No | Fair |
| e-Ref #6 | Yes | Yes | No | Yes | No | Yes | Yes | No | Yes | Yes | Yes | Yes | Yes | No | Good |
| e-Ref #7 | Yes | Yes | Yes | No | No | No | Not applicable | No | Yes | No | Yes | Yes | Yes | No | Fair |
| e-Ref #11 | Yes | No | Yes | Yes | No | No | Not applicable | No | No | No | Yes | No | Not applicable | No | Poor |
| e-Ref #17 | No | No | Yes | Yes | No | No | Not applicable | No | No | No | Yes | No | Not applicable | No | Poor |
| e-Ref #22 | Yes | Yes | Yes | Yes | No | No | Not applicable | No | Yes | No | Yes | Yes | Yes | Yes | Fair |
| e-Ref #26 | Yes | Yes | No | Yes | No | Yes | Yes | No | Yes | No | Yes | No | Yes | No | Fair |
| e-Ref #27 | Yes | No | No | Yes | No | No | Not applicable | Yes | No | No | Yes | No | Not applicable | No | Poor |
| e-Ref #28 | Yes | Yes | Yes | No | No | Yes | Yes | No | Yes | Yes | Yes | No | Yes | No | Fair |
| e-Ref #29 | Yes | Yes | Yes | No | No | Yes | Yes | No | Yes | No | Yes | Yes | No | No | Fair |
| e-Ref #33 | Yes | No | Yes | Yes | No | No | Not applicable | No | Yes | No | Yes | No | Not applicable | No | Fair |
| e-Ref #34 | Yes | Yes | No | Yes | No | Yes | Yes | Not applicable | Yes | No | Yes | No | No | No | Fair |
| e-Ref #35 | Yes | Yes | Yes | Yes | No | Yes | Yes | No | Yes | Yes | Yes | Yes | Yes | No | Good |
| e-Ref #36 | Yes | Yes | Yes | Yes | No | No | Not applicable | No | Yes | No | Yes | No | Not applicable | No | Fair |

Q1: Was the research question or objective in this paper clearly stated?

Q2: Was the study population clearly specified and defined?

Q3: Was the participation rate of eligible persons at least 50%?

Q4: Were all the subjects selected or recruited from the same or similar populations (including the same time period)? Were inclusion and exclusion criteria for being in the study prespecified and applied uniformly to all participants?

Q5: Was a sample size justification, power description, or variance and effect estimates provided?

Q6: For the analyses in this paper, were the exposure(s) of interest measured prior to the outcome(s) being measured?

Q7: Was the timeframe sufficient so that one could reasonably expect to see an association between exposure and outcome if it existed?

Q8: For exposures that can vary in amount or level, did the study examine different levels of the exposure as related to the outcome (e.g., categories of exposure, or exposure measured as continuous variable)?

Q9: Were the exposure measures (independent variables) clearly defined, valid, reliable, and implemented consistently across all study participants?

Q10: Was the exposure(s) assessed more than once over time?

Q11: Were the outcome measures (dependent variables) clearly defined, valid, reliable, and implemented consistently across all study participants?

Q12: Were the outcome assessors blinded to the exposure status of participants?

Q13: Was loss to follow-up after baseline 20% or less?

Q14: Were key potential confounding variables measured and adjusted statistically for their impact on the relationship between exposure(s) and outcome(s)?

**Supplementary Table 4: Quality appraisals for case-control studies using the NHLBI study quality assessment tool.**

| **Study** | **Q1** | **Q2** | **Q3** | **Q4** | **Q5** | **Q6** | **Q7** | **Q8** | **Q9** | **Q10** | **Q11** | **Q12** | **Overall quality rating** |
| --- | --- | --- | --- | --- | --- | --- | --- | --- | --- | --- | --- | --- | --- |
| e-Ref #1 | Yes | Yes | No | Yes | Yes | Yes | No | No | Yes | Yes | No | Yes | Fair |
| e-Ref #4 | Yes | Yes | No | Yes | Yes | Yes | No | No | Yes | Yes | No | Yes | Fair |
| e-Ref #5 | No | Yes | No | Yes | No | Yes | No | No | No | No | No | No | Poor |
| e-Ref #9 | Yes | Yes | No | No | Yes | Yes | No | No | No | Yes | No | Yes | Fair |
| e-Ref #10 | Yes | Yes | No | Yes | Yes | Yes | No | No | Yes | Yes | No | No | Fair |
| e-Ref #12 | Yes | Yes | No | Yes | Yes | Yes | Yes | No | Yes | Yes | Yes | Yes | Good |
| e-Ref #13 | Yes | Yes | No | No | Yes | Yes | No | No | Yes | Yes | Yes | No | Fair |
| e-Ref #15 | Yes | Yes | No | Yes | Yes | Yes | Yes | No | No | Yes | Yes | No | Fair |
| e-Ref #16 | Yes | Yes | No | Yes | Yes | Yes | Yes | No | No | Yes | No | No | Fair |
| e-Ref #19 | Yes | Yes | No | Yes | Yes | Yes | No | No | No | Yes | Yes | No | Fair |
| e-Ref #20 | Yes | Yes | No | No | Yes | Yes | No | No | No | Yes | Yes | No | Fair |
| e-Ref #23 | Yes | No | No | Yes | No | Yes | No | No | No | No | No | No | Poor |
| e-Ref #24 | Yes | No | No | No | No | Yes | Yes | No | No | No | Yes | No | Poor |
| e-Ref #25 | Yes | Yes | No | Yes | Yes | Yes | No | No | No | Yes | Yes | No | Fair |
| e-Ref #30 | Yes | Yes | No | Yes | Yes | Yes | No | No | No | Yes | No | No | Fair |
| e-Ref #31 | Yes | Yes | No | No | Yes | Yes | No | No | No | Yes | No | No | Fair |
| e-Ref #32 | Yes | Yes | No | Yes | Yes | Yes | No | No | No | Yes | Yes | No | Fair |
| e-Ref #37 | Yes | Yes | No | Yes | Yes | Yes | No | No | No | Yes | Yes | No | Fair |

Q1: Was the research question or objective in this paper clearly stated and appropriate?

Q2: Was the study population clearly specified and defined?

Q3: Did the authors include a sample size justification?

Q4: Were controls selected or recruited from the same or similar population that gave rise to the cases (including the same timeframe)?

Q5: Were the definitions, inclusion and exclusion criteria, algorithms or processes used to identify or select cases and controls valid, reliable, and implemented consistently across all study participants?

Q6: Were the cases clearly defined and differentiated from controls?

Q7: If less than 100 percent of eligible cases and/or controls were selected for the study, were the cases and/or controls randomly selected from those eligible?

Q8: Was there use of concurrent controls?

Q9: Were the investigators able to confirm that the exposure/risk occurred prior to the development of the condition or event that defined a participant as a case?

Q10: Were the measures of exposure/risk clearly defined, valid, reliable, and implemented consistently (including the same time period) across all study participants?

Q11: Were the assessors of exposure/risk blinded to the case or control status of participants?

Q12: Were key potential confounding variables measured and adjusted statistically in the analyses? If matching was used, did the investigators account for matching during study analysis?

**Supplementary Table 5: Quality appraisals for case-series using the NHLBI study quality assessment tool.**

| **Study** | **Q1** | **Q2** | **Q3** | **Q4** | **Q5** | **Q6** | **Q7** | **Q8** | **Q9** | **Overall quality rating** |
| --- | --- | --- | --- | --- | --- | --- | --- | --- | --- | --- |
| e-Ref #18 | Yes | Yes | Yes | No | Not applicable | Yes | No | Not applicable | Yes | Good |
| e-Ref #21 | Yes | Yes | No | No | Not applicable | Yes | No | Not applicable | Yes | Fair |

Q1: Was the study question or objective clearly stated?

Q2: Was the study population clearly and fully described, including a case definition?

Q3: Were the cases consecutive?

Q4: Were the subjects comparable?

Q5: Was the intervention clearly described?

Q6: Were the outcome measures clearly defined, valid, reliable, and implemented consistently across all study participants?

Q7: Was the length of follow-up adequate?

Q8: Were the statistical methods well-described?

Q9: Were the results well-described?

**Supplementary Table 6: Echocardiography parameters reported in meta-analysis.**

| Study | EF | E/A | IVSd | LVEDD | LVESD | E | A | LVPWd | FS | IVRT | e' | E/e' | LV Mass | LA Diameter | DT | LVEDV | LA volume |
| --- | --- | --- | --- | --- | --- | --- | --- | --- | --- | --- | --- | --- | --- | --- | --- | --- | --- |
| e-Ref #1, 2020 | Y |  |  |  |  |  |  |  | Y |  |  |  |  |  |  |  |  |
| e-Ref #4, 2021 | Y | Y |  | Y | Y | Y | Y |  |  |  | Y |  | Y | Y |  | Y | Y |
| e-Ref #9, 2013 | Y | Y | Y | Y | Y |  |  | Y | Y |  |  |  | Y | Y |  |  |  |
| e-Ref #10, 2014 | Y | Y | Y | Y | Y | Y | Y | Y | Y | Y | Y | Y | Y |  | Y | Y |  |
| e-Ref #13, 2013 | Y | Y | Y | Y | Y | Y | Y | Y | Y | Y | Y |  |  | Y | Y | Y | Y |
| e-Ref #15, 2018 | Y | Y | Y |  |  | Y | Y | Y | Y | Y | Y | Y | Y |  | Y |  |  |
| e-Ref #20, 2019 |  |  |  |  |  |  |  |  |  | Y |  | Y |  |  |  |  |  |
| e-Ref #30, 2020 | Y |  | Y | Y | Y | Y | Y | Y |  |  |  |  |  | Y |  |  |  |
| e-Ref #31, 2018 | Y | Y | Y | Y | Y | Y | Y |  |  | Y | Y | Y | Y | Y | Y | Y | Y |
| e-Ref #37, 2021 | Y | Y | Y | Y | Y | Y | Y | Y | Y | Y | Y | Y |  |  | Y |  |  |
| Total (n) | 9 | 7 | 7 | 7 | 7 | 7 | 7 | 6 | 6 | 6 | 6 | 5 | 5 | 5 | 5 | 4 | 3 |

EF= left ventricle ejection fraction; E/A= the peak velocities of early (E)/late (A) mitral wave velocity ratio; IVSd= interventricular septum diameter at end-diastole; LVEDD= left ventricular end-diastolic diameter; LVESD= left ventricular end-systolic diameter; E=E-wave velocity; A=A-wave velocity; LVPWd= LV posterior wall thickness at end-diastole; FS=fractional shortening; e’= lateral peak early diastolic annular (e’) wave velocity; LA= left atrium; DT= E-wave deceleration time; LVEDV= LV end-diastolic volume.

**Supplementary Table 7: Additional meta-analysis results of echocardiography parameters.**

| **Echo parameter** | Mean Difference | 95% CI Lower | 95% CI Higher | p | Heterogeneity I^2^ (%) |
| --- | --- | --- | --- | --- | --- |
| ***Left ventricular functional echocardiogram parameters*** |  |  |  |  |  |
| Fractional Shortening (%) | -1.33 | -3.80 | 1.13 | 0.289 | 90.06 |
| E wave velocity (cm/s) | 5.92 | -2.64 | 14.48 | 0.176 | 96.58 |
| E/A ratio | 0.03 | -0.16 | 0.21 | 0.779 | 96.97 |
| E wave deceleration time (ms) | 11.68 | -5.19 | 28.56 | 0.175 | 94.70 |
| e' wave velocity (cm/s) | -0.42 | -1.50 | 0.65 | 0.289 | 90.06 |
| ***Structural echocardiogram parameters*** |  |  |  |  |  |
| Left ventricular end-diastole volume (mL) | 2.59 | -2.17 | 7.35 | 0.286 | 40.36 |
| Left ventricular end-diastolic diameter (cm) | 0.07 | -0.07 | 0.21 | 0.356 | 80.99 |
| Left ventricular end-systolic diameter (cm) | 0.11 | -0.04 | 0.26 | 0.140 | 89.19 |
| Interventricular septum diameter at end-diastole (cm) | 0.03 | -0.01 | 0.06 | 0.183 | 67.22 |
| Left ventricular posterior wall thickness at end-diastole (cm) | -0.01 | -0.04 | 0.02 | 0.527 | 45.84 |
| Left ventricle mass index (mg/m^2^) | 3.62 | -2.01 | 9.25 | 0.208 | 68.37 |
| Left atrium diameter (cm) | 0.10 | -0.07 | 0.26 | 0.258 | 82.82 |

CI=confidence interval.

**Supplementary Table 8: Random-effects meta-regression of echocardiography parameters against potential effect moderators for the associated between cardiac functional/structural changes and epilepsy.**

| **Echo parameter** | **Age (years)** | | | | | | |
| --- | --- | --- | --- | --- | --- | --- | --- |
| *Left ventricular functional echocardiogram parameters* | Study (n) | Coef | p>[z] | 95% CI Lower | 95% CI Higher | R^2^ (% heterogeneity accounted for) | I^2^ (% residual heterogeneity) |
| Ejection Fraction | 9 | -0.072 | 0.413 | -0.251 | 0.107 | 0 | 87.56 |
| E/A ratio | 7 | 0.012 | 0.157 | -0.005 | 0.030 | 14.52 | 96.45 |
| E wave velocity | 7 | 0.563 | 0.139 | -23.533 | 11.591 | 17.1 | 94.45 |
| A wave velocity | 7 | -0.285 | 0.010* | -0.50 | -0.07 | 64.07 | 35.89 |
| E wave deccelration time | 5 | 0.755 | 0.517 | -1.528 | 3.039 | 0 | 95.47 |
| Lateral E wave velocity | 6 | 0.082 | 0.073 | -0.008 | 0.171 | 36.67 | 71.77 |
| Isovolumetric Relaxation Time | 6 | -0.437 | 0.434 | -1.532 | 0.658 | 0 | 97.85 |
| Fractional Shortening | 6 | -0.265 | 0.035* | -0.511 | -0.019 | 45.14 | 82.19 |
| E/e' ratio | 5 | -0.030 | 0.255 | -0.082 | 0.022 | 8.46 | 78.92 |
| *Structural echocardiogram parameters* |  |  |  |  |  |  |  |
| LA Diameter | 5 | -0.002 | 0.936 | 0.044 | 0.041 | 0 | 87.19 |
| LV end-diastole diamter | 7 | -0.006 | 0.411 | -0.021 | 0.009 | 0 | 81.87 |
| LV end-systole diameter | 7 | -0.009 | 0.228 | -0.024 | 0.006 | 7.12 | 88.07 |
| LV Posterior Wall thickness | 6 | -0.002 | 0.272 | -0.005 | 0.001 | 18.6 | 40.61 |
| Interventricular Septum Diameter at End Diastole | 7 | 0.001 | 0.583 | -0.003 | 0.006 | 0 | 68.4 |
| LV Mass index | 5 | -0.136 | 0.590 | -0.632 | 0.359 | 0 | 69.7 |
|  |  |  |  |  |  |  |  |
| **Echo parameter** | **Sex (% female)** | | | | | | |
| *Left ventricular functional echocardiogram parameters* | Study (n) | Coef | p>[z] | 95% CI Lower | 95% CI Higher | R^2^ (% heterogeneity accounted for) | I^2^ (% residual heterogeneity) |
| Ejection Fraction | 9 | -1.717 | 0.829 | -17.315 | 13.881 | 0 | 89.66 |
| E/A ratio | 7 | 0.608 | 0.406 | -0.827 | 2.043 | 0 | 95.71 |
| E wave velocity | 7 | 3.303 | 0.930 | -69.904 | 76.511 | 0 | 95.44 |
| A wave velocity | 7 | -6.660 | 0.567 | -29.46 | 16.14 | 0 | 62.17 |
| E wave deccelration time | 5 | -62.34 | 0.325 | -186.412 | 61.726 | 0 | 91.46 |
| Lateral E wave velocity | 6 | 1.437 | 0.744 | -7.175 | 10.049 | 0 | 79.52 |
| Isovolumetric Relaxation Time | 6 | -37.552 | 0.209 | -96.135 | 21.032 | 10.34 | 88.38 |
| Fractional Shortening | 6 | -1.878 | 0.882 | -26.716 | 22.96 | 0 | 92.33 |
| E/e' ratio | 5 | 0.073 | 0.956 | -2.514 | 2.659 | 0 | 82.01 |
| *Structural echocardiogram parameters* |  |  |  |  |  |  |  |
| LA Diameter | 5 | -1.023 | 0.076 | -2.155 | 0.108 | 37.78 | 72.23 |
| LV end-diastole diamter | 7 | 0.342 | 0.541 | -0.659 | 0.456 | 0 | 82.45 |
| LV end-systole diameter | 7 | 0.176 | 0.771 | -1.013 | 1.365 | 0 | 90.79 |
| LV Posterior Wall thickness | 6 | 0.067 | 0.638 | -0.213 | 0.348 | 0 | 49.77 |
| Interventricular Septum Diameter at End Diastole | 7 | -0.08 | 0.592 | -0.373 | 0.213 | 0 | 68.66 |
| LV Mass index | 5 | -2.207 | 0.909 | -40.109 | 35.695 | 0 | 66.64 |
|  |  |  |  |  |  |  |  |
| **Echo parameter** | **BMI (kg/m^2^)** | | | | | | |
| *Left ventricular functional echocardiogram parameters* | Study (n) | Coef | p>[z] | 95% CI Lower | 95% CI Higher | R^2^ (% heterogeneity accounted for) | I^2^ (% residual heterogeneity) |
| Ejection Fraction | 6 | 0.071 | 0.762 | 30.387 | 0.529 | 0 | 93.51 |
| E/A ratio | 5 | 0.024 | 0.122 | -0.006 | 0.055 | 25.74 | 93.08 |
| E wave velocity | 5 | 0.5 | 0.566 | -1.21 | 2.21 | 0 | 95.19 |
| A wave velocity | 5 | -0.346 | 0.155 | -0.82 | 0.13 | 22.82 | 47.73 |
| *Structural echocardiogram parameters* |  |  |  |  |  |  |  |
| LA Diameter | 5 | 0.009 | 0.625 | -0.028 | 0.046 | 0 | 86.08 |
| LV end-diastole diamter | 6 | -0.009 | 0.542 | -0.037 | 0.019 | 0 | 85.35 |
| LV end-systole diameter | 6 | -0.021 | 0.082 | -0.046 | 0.003 | 29.98 | 87.37 |
| Interventricular Septum Diameter at End Diastole | 5 | -0.114 | 0.286 | -0.385 | 0.157 | 4.06 | 70.38 |
|  |  |  |  |  |  |  |  |
| **Echo parameter** | **Patients with Generalised Epilepsy (%)** | | | | | | |
| *Left ventricular functional echocardiogram parameters* | Study (n) | Coef | p>[z] | 95% CI Lower | 95% CI Higher | R^2^ (% heterogeneity accounted for) | I^2^ (% residual heterogeneity) |
| Ejection Fraction | 6 | -3.863 | 0.226 | -10.122 | 2.395 | 9.73 | 83.52 |
| E/A ratio | 6 | 0.223 | 0.474 | -0.388 | 0.834 | 0 | 97.65 |
| E wave velocity | 6 | 14.416 | 0.278 | -11.616 | 40.449 | 3.32 | 96.49 |
| A wave velocity | 6 | -4.794 | 0.237 | -12.738 | 3.15 | 10.23 | 54.73 |
| E wave deccelration time | 5 | 12.608 | 0.665 | -44.437 | 69.653 | 0 | 95.43 |
| Lateral E wave velocity | 6 | 2.247 | 0.096 | -0.397 | 4.892 | 30.51 | 73.38 |
| Isovolumetric Relaxation Time | 6 | -14.489 | 0.238 | -38.549 | 9.572 | 7.22 | 97.07 |
| E/e' ratio | 5 | -0.505 | 0.382 | -1.638 | 0.627 | 0 | 80.87 |
| *Structural echocardiogram parameters* |  |  |  |  |  |  |  |
| LV end-diastole diamter | 5 | -0.018 | 0.895 | -0.293 | 0.256 | 0 | 32.32 |
| LV end-systole diameter | 5 | 0.175 | 0.432 | -0.261 | 0.611 | 0 | 83.72 |
| Interventricular Septum Diameter at End Diastole | 5 | -0.021 | 0.697 | -0.129 | 0.086 | 0 | 38.39 |
|  |  |  |  |  |  |  |  |
| **Echo parameter** | **Year since Epilepsy Diagnosed (years)** | | | | | | |
| *Left ventricular functional echocardiogram parameters* | Study (n) | Coef | p>[z] | 95% CI Lower | 95% CI Higher | R^2^ (% heterogeneity accounted for) | I^2^ (% residual heterogeneity) |
| Ejection Fraction | 6 | 0.192 | 0.262 | -0.143 | 0.527 | 5.38 | 89.20 |
| E/A ratio | 5 | 0.003 | 0.818 | -0.022 | 0.028 | 0 | 93.16 |
| E wave velocity | 6 | -0.128 | 0.85 | -1.454 | 1.198 | 0 | 95.96 |
| A wave velocity | 6 | -0.273 | 0.171 | -0.665 | 0.118 | 19.68 | 43.69 |
| Lateral E wave velocity | 5 | 0.013 | 0.889 | -0.172 | 0.198 | 0 | 84.73 |
| *Structural echocardiogram parameters* |  |  |  |  |  |  |  |
| LV end-diastole diamter | 5 | -0.007 | 0.29 | -0.019 | 0.006 | 43.33 | 13.11 |
| LV end-systole diameter | 5 | -0.020 | 0.041* | -0.038 | 0 | 49.61 | 79.74 |
| Interventricular Septum Diameter at End Diastole | 5 | 0.009 | 0.053 | 0.000 | 0.017 | 47.91 | 62.67 |
|  |  |  |  |  |  |  |  |
| **Echo parameter** | **Patients with Monotherapy (%)** | | | | | | |
| *Left ventricular functional echocardiogram parameters* | Study (n) | Coef | p>[z] | 95% CI Lower | 95% CI Higher | R^2^ (% heterogeneity accounted for) | I^2^ (% residual heterogeneity) |
| Ejection Fraction | 5 | -0.601 | 0.65 | -3.195 | 1.993 | 0 | 38.47 |

BMI=Body mass index; CI=confidence interval; *, p<0.05.

**Supplementary Table 9: Characteristics of studies reporting the effect of epilepsy treatments on echocardiography findings.**

| **Study** | **Study design** | **Population size (n)** | **Age group** | **Age (years), mean ± SD** | **Female sex, n (%)** | **BMI (kg/m^2^), mean ± SD** | **Population** | **Treatment** | **Dosage** | **Treatment duration** | **Cardiac findings** |
| --- | --- | --- | --- | --- | --- | --- | --- | --- | --- | --- | --- |
| e-Ref #15, 2018 | Cohort study, prospective | 40 | Pediatric | 10.03 ± 3.75 | 57.5% | NR | Idiopathic epilepsy | VPA | 10 mg/kg/day - 20mg/kg/day | 6 months | No difference between groups |
| e-Ref #26, 2009 | Cohort study, prospective | 40 | Pediatric | 9.5 ± 2.8 | 22.5% | NR | Epilepsy | CBZ | 5 mg/kg/day - 10mg/kg/day | 12 months | No difference between groups |
| e-Ref #28, 2016 | Cohort study, prospective | 61 | Pediatric | 5.4 ± 3.3 | 47.50% | 16.4 (11–29)^a^ | Intractable epilepsy | Ketogenic diet | 3:1 ratio of fat to carbohydrates plus protein | 13 months | No differences between groups |
| e-Ref #29, 2020 | Cohort study, prospective | 18 | Pediatric | 0.675 ± 0.3 | 27.80% | NR | Infantile spasm | ACTH | 0.5 mg/kg < 10kg;  1 mg/kg >10 kg | 2 months | No differences to echocardiography between groups. However, mild septal hypertrophy and increased left ventricle mass index with ACTH treatment; changes reversed after discontinuation of treatment |
| e-Ref #32, 2013 | Case-control study, prospective | 43  (23 epilepsy with treatment, 20 epilepsy no treatment) | Pediatric and adult | Treatment (11 ± 8), control (10 ± 7) | Treatment 52.2%, control 55.0% | Treatment (20 ± 2), control (21 ± 3) | Epilepsy | Ketogenic diet | fat/protein + carbohydrate ratio 4:1 | ≥6 months | No differences to echocardiography between groups. However, measures of arterial stiffness and serum cholesterol and triglycerides were increased in the treated group |
| e-Ref #34, 2015 | Cohort study, prospective | 32 | Pediatric | 3.79^a^ | 42.20% | 15.9 ± 2.3 | Refractory epilepsy | ketogenic diet | standardized 3:1 ketogenic diet | 6 months | Decreased e' velocity and e'/a' ratio in the treated group, potentially suggestive of right ventricular diastolic dysfunction |
| e-Ref #35, 2010 | Cohort study, prospective | 10 | Pediatric and adult | 32.4 ± 9.1 | 60% | NR | Refractory epilepsy | VNS | NA | 7.7 ± 2.3 months | No differences to echocardiography between groups. However, systolic and diastolic BP were increased post-VNS |

a’=Late diastolic myocardial velocity; ACTH=Adrenocorticotropic hormone; BMI=Body mass index; BP=Blood pressure; CBZ=Carbamazepine; e’/a’ ratio=early diastolic myocardial velocity/late diastolic myocardial velocity; LV=Left ventricular; LVEF=Ejection fraction; FS=Fractional shortening; NR=Not reported; VNS=Vagus nerve stimulation; VPA=Valproic acid.

a: Data was reported as median (range).

**eReferences**

1. Schreiber JM, Frank LH, Kroner BL, Bumbut A, Ismail MO, Gaillard WD. Children with refractory epilepsy demonstrate alterations in myocardial strain. Epilepsia 2020;61:2234-2243.

2. Cihan E, Devinsky O, Hesdorffer DC, et al. Temporal trends and autopsy findings of SUDEP based on medico-legal investigations in the United States. Neurology 2020;95:e867-e877.

3. Karlovich E, Devinsky O, Brandsoy M, Friedman D. SUDEP among young adults in the San Diego County Medical Examiner Office. Epilepsia 2020;61:e17-e22.

4. Fialho GL, Wolf P, Walz R, Lin K. Left ventricle end-systolic elastance, arterial-effective elastance, and ventricle-arterial coupling in Epilepsy. Acta Neurol Scand 2021;143:34-38.

5. Eskandarian R, Asghari N, Darban M, Ghorbani R. Cardiac troponin levels following complicated and uncomplicated epileptic seizures. Arch Med Res 2011;42:439-442.

6. Kulhas Celik I, Tasdemir HA, Ince H, Celik H, Sungur M. Evaluation ofserum free carnitine/acylcarnitine levels and left ventricular systolic functions in children with idiopathic epilepsy receiving valproic acid. Clin Neurol Neurosurg 2018;170:106-112.

7. Belcour D, Jabot J, Grard B, et al. Prevalence and Risk Factors of Stress Cardiomyopathy After Convulsive Status Epilepticus in ICU Patients. Crit Care Med 2015;43:2164-2170.

8. Zhuo L, Zhang Y, Zielke HR, et al. Sudden unexpected death in epilepsy: Evaluation of forensic autopsy cases. Forensic Sci Int 2012;223:171-175.

9. M MR, El-Shahat N, A AO, et al. Interictal electrocardiographic and echocardiographic changes in patients with generalized tonic-clonic seizures. Int Heart J 2013;54:171-175.

10. Kibar AE, Unver O, Oflaz MB, et al. Effect of antiepilepsy drug therapy on ventricular function in children with epilepsy: a tissue Doppler imaging study. Pediatr Cardiol 2014;35:280-288.

11. Stecker EC, Reinier K, Uy-Evanado A, et al. Relationship Between Seizure Episode and Sudden Cardiac Arrest in Patients With Epilepsy A Community-Based Study. Circ-Arrhythmia Elec 2013;6:912-916.

12. Bardai A, Blom MT, van Noord C, Verhamme KM, Sturkenboom MCJM, Tan HL. Sudden cardiac death is associated both with epilepsy and with use of antiepileptic medications. Heart 2015;101:17-22.

13. Bilgi M, Yerdelen D, Colkesen Y, Muderrisoglu H. Evaluation of left ventricular diastolic function by tissue Doppler imaging in patients with newly diagnosed and untreated primary generalized epilepsy. Seizure-European Journal of Epilepsy 2013;22:537-541.

14. Manno EM, Pfeifer EA, Cascino GD, Noe KH, Wijdicks EFM. Cardiac pathology in status epilepticus. Ann Neurol 2005;58:954-957.

15. Celik SF, Baratali E, Guven AS, Torun YA. Left ventricular myocardial deformation abnormalities in seizure-free children with epilepsy. Seizure-European Journal of Epilepsy 2018;61:153-157.

16. Davis GG, McGwin G. Comparison of heart mass in seizure patients dying of sudden unexplained death in epilepsy to sudden death due to some other cause. Am J Foren Med Path 2004;25:23-28.

17. Esen Melez I, Arslan MN, Melez DO, Sanli AN, Koc S. Sudden Unexpected Death in Epilepsy: A Retrospective Autopsy Study of 112 Epileptic Patients. Noropsikiyatri Ars 2017;54:225-233.

18. Falconer B, Rajs J. Post-mortem findings of cardiac lesions in epileptics: a preliminary report. Forensic Sci 1976;8:63-71.

19. El Amrousy D, Abd El-Hafez M, Nashat M, Hodeib H. Cardiac injury after convulsive status epilepticus in children. Eur J Paediatr Neurol 2017;21:648-653.

20. Tosun O, Karatoprak E. Analysis of tissue Doppler parameters and 24-hour heart rate variations in children with newly diagnosed untreated idiopathic epilepsy in interictal period. Epilepsy & Behavior 2019;90:11-14.

21. Dasheiff RM. Sudden Unexpected Death in Epilepsy - a Series from an Epilepsy Surgery Program and Speculation on the Relationship to Sudden Cardiac Death. J Clin Neurophysiol 1991;8:216-222.

22. Devinsky O, Kim A, Friedman D, Bedigian A, Moffatt E, Tseng ZH. Incidence of cardiac fibrosis in SUDEP and control cases. Neurology 2018;91:E55-E61.

23. Vesterby A, Gregersen M, Baandrup U. The myocardium in epileptics. Am J Forensic Med Pathol 1986;7:288-290.

24. Natelson BH, Suarez RV, Terrence CF, Turizo R. Patients with epilepsy who die suddenly have cardiac disease. Arch Neurol-Chicago 1998;55:857-860.

25. P-Codrea S, Dalager-Pedersen S, Baandrup U, Dam M, Vesterby-Charles A. Sudden unexpected death in epilepsy - Is death by seizures a cardiac disease? Am J Foren Med Path 2005;26:99-105.

26. Arhan E, Aycicek S, Akalin N, Guven A, Kose G. Cardiac Effects of Carbamazepine Treatment in Childhood Epilepsy. Neurologist 2009;15:268-273.

27. Hajsadeghi S, Afisharian S, Fereshtehnejad SM, Keramati MR, Mollahoseini R. Serum Levels of Cardiac Troponin I in Patients with Uncomplicated Epileptic Seizure. Archives of Medical Research 2009;40:24-28.

28. Ozdemir R, Kucuk M, Guzel O, Karadeniz C, Yilmaz U, Mese T. Does ketogenic diet have any negative effect on cardiac systolic and diastolic functions in children with intractable epilepsy?: One-year follow-up results. Brain Dev-Jpn 2016;38:842-847.

29. Kutluk G, Ekici F, Turan O, Bektas O, Kadem N. Effects of Adrenocorticotropic Hormone Treatment on Heart Muscle in Patients with Infantile Spasm. Cureus 2020;12.

30. Asoglu R, Ozdemir M, Aladag N, Asoglu E. Evaluation of Cardiac Repolarization Indices in Epilepsy Patients Treated with Carbamazepine and Valproic Acid. Medicina-Lithuania 2020;56.

31. Genc F, Genc A, Kucukseymen E, et al. Evaluation of systolic and diastolic cardiac functions and heart rate variability in patients with juvenile myoclonic epilepsy. Cesk Slov Neurol N 2018;81:699-705.

32. Coppola G, Natale F, Torino A, et al. The impact of the ketogenic diet on arterial morphology and endothelial function in children and young adults with epilepsy: A case-control study. Seizure-European Journal of Epilepsy 2014;23:260-265.

33. Earnest MP, Thomas GE, Eden RA, Hossack KF. The Sudden Unexplained Death Syndrome in Epilepsy - Demographic, Clinical, and Postmortem Features. Epilepsia 1992;33:310-316.

34. Doksoz O, Celegen K, Guzel O, et al. The Short-Term Effects of Ketogenic Diet on Cardiac Ventricular Functions in Epileptic Children. Pediatr Neurol 2015;53:233-237.

35. Cadeddu C, Deidda M, Mercuro G, et al. Cardiovascular modulation during vagus nerve stimulation therapy in patients with refractory epilepsy. Epilepsy Research 2010;92:145-152.

36. Chahal CAA, Tester DJ, Fayyaz AU, et al. Confirmation of Cause of Death Via Comprehensive Autopsy and Whole Exome Molecular Sequencing in People With Epilepsy and Sudden Unexpected Death. Journal of the American Heart Association 2021;10:e021170-e021170.

37. Ibrahim A, Soliman WM, Mesbah BE-DM, Salem AS. Left ventricular dysfunction and cardiac autonomic imbalance in children with drug-resistant epilepsy. Epilepsy Research 2021;176.
